# An outcomes analysis of anterior epistaxis management in the emergency department

**DOI:** 10.1186/s40463-016-0138-2

**Published:** 2016-04-11

**Authors:** E. Newton, A. Lasso, W. Petrcich, S. J. Kilty

**Affiliations:** University of Ottawa, Ottawa, ON Canada; Department of Otolaryngology - Head and Neck Surgery, University of Ottawa, Ontario, Canada; Ottawa Hospital Research Institute (OHRI), Ottawa, ON Canada

**Keywords:** Epistaxis, Treatment, Anterior epistaxis, Tertiary care, Emergency department

## Abstract

**Background:**

Many treatment options exist for the management of anterior epistaxis. However, little is known about treatment outcomes. The objective was to identify the currently utilised methods of management and outcomes for patients with anterior epistaxis presenting to the emergency department (ED) at a Canadian tertiary care center.

**Methods:**

A retrospective review of ED visits from January 2012-May 2014 for adult patients with a diagnosis of anterior epistaxis was performed. Patient demographic data, comorbidities, and treatment methods were documented. The effectiveness of different treatment modalities was determined.

**Results:**

Three hundred fifty-three primary anterior epistaxis cases were included. Mean patient age was 70 years and 49 % of patients were female. Comorbidities included hypertension (56 %), diabetes (19 %), CAD (28 %), and atrial fibrillation (27 %). A large proportion of the cohort (61 %) was on at least one anticoagulant or antiplatelet therapy. The most common utilised treatment modalities were silver nitrate cauterization, Merocel®, petroleum gauze packing, nasal clip and 15 % were simply observed. Initial treatment success was achieved in 74 % of cases. Of patients receiving specific treatment modalities, silver nitrate cauterization had the highest success rate at 80 %. 26 % of patients returned to the ED for recurrence of epistaxis with highest rates occurring in the nasal clip (59 %), Merocel® (26 %), and petroleum gauze packing (42 %) groups.

**Conclusions:**

The differences in recurrence rate among the different treatment modalities observed may be due to true differences in effectiveness or differences in treatment selection by the ED physicians based on severity of epistaxis. Cauterization with silver nitrate, however, offers the added benefit of no need for follow up. Further study is needed to elucidate the most efficacious treatment modality based on epistaxis severity.

## Background

Epistaxis, is an exceedingly common presenting problem to hospitals in North America accounting for approximately 1 in 200 emergency department (ED) visits in the United States [1]. Although difficult to truly assess, it has been estimated that 60 % of the population has at least 1 episode of epistaxis in their lifetime of which 6 % seek medical treatment [2]. The sheer incidence of epistaxis constitutes it as an important condition in terms of cost, time and resource management. Thus, it is important to identify the most efficacious treatment modality in the realms of treatment success.

There are many treatment modalities and algorithms for epistaxis described in the literature [3–9]. Most approaches describe initiating packing and nasal pressure and escalating to more invasive and time consuming treatments if that fails. For anterior epistaxis there is evidence for the use of chemical cautery [10], anterior packing [5], and other hemostatic matrices [4]. All of these modalities have been shown to have good efficacy in achieving hemostasis. However, there is insufficient literature evaluating these modalities and their effectiveness when utilised in the ED. Further, at this time there are no widely accepted treatment guidelines and treatment selection is a matter of individual ED physician preference.

### Importance

Considering that anterior epistaxis is a very common and treatable condition it is important to optimize efficiency and effectiveness when treating this disorder. Although, there is evidence for each individual treatment modality, the literature is deficient as to current ED physician practices and the outcomes for use of the many modalities.

### Goals of this investigation

The purpose of this study was twofold, first to assess the current practices utilised in a Canadian Tertiary Care center for anterior epistaxis management and second, to evaluate the outcomes of these treatments.

## Methods

### Study design and setting

With the approval of the Research Ethics Board at the Ottawa Hospital Research Institute a retrospective review of all patient visits to the ED at The Ottawa Hospital (TOH), a Canadian tertiary care center, with a primary diagnosis of anterior epistaxis during the period of January 2012 to May 2014 was performed.

### Selection of participants

**Fig. 1:**
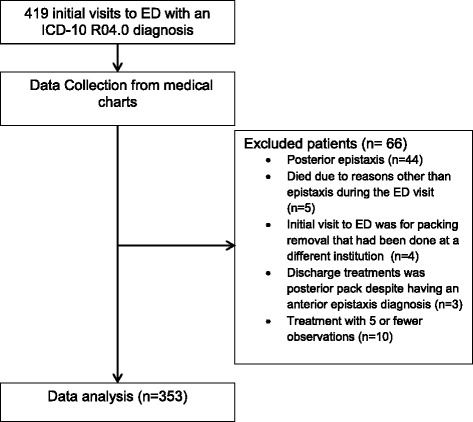
Study flowchart

Adult patients with a primary diagnosis of epistaxis in the emergency department were included in this study. Records were identified by the health records department using the ICD-10 code for epistaxis (R04-0). The epistaxis codes do not differentiate between anterior and posterior epistaxis; thus all records were hand searched and patients with a diagnosis of posterior epistaxis or concurrent anterior and posterior epistaxis were excluded. Patients that presented with epistaxis due to a complication of pre-existing conditions such as end stage cancer were excluded. Patients who died during the ED visit for reasons other than epistaxis were also excluded. Patients with an initial visit to the ED for packing removal that had been placed at a different institution and patients who were treated as posterior epistaxis despite having an anterior epistaxis diagnosis were also excluded. Patients who received treatment with a modality that was used in five or fewer cases were also excluded from analysis. See Fig. 1 for study flow chart.

### Methods and measurements

From the identified charts data was abstracted including patient demographics, comorbidities, the treatment modalities used, course in the emergency department, admission, concurrent medical disorders, medications and finally recurrence or ED follow-up information. Treatment modalities identified for data abstraction included conservative (no treatment), nasal clip, petroleum gauze packing, Merocel® packing, Floseal®, Surgicel®, Epistat®, silver nitrate cautery, electrocautery, endoscopic surgery, arterial embolization and other treatments not otherwise specified (NOS). The “other packing” group in this study received anterior petroleum gauze packing or equivalent.

### Outcomes

For each treatment modality, success was defined as patients who were diagnosed with anterior epistaxis, who received treatment and did not present with a recurrence within 14 days of their original date of presentation [11]. Conversely, failure was defined as the patients who had an ipsilateral recurrence of epistaxis within 14 days of initial treatment. The treatment type was recorded based on the treatment modality used to arrest the bleeding that led to the patient’s discharge from the ED. Follow-up was defined as patients who were administered a specific treatment and who were subsequently booked and received follow-up care in the ED for either packing removal or to check the site of epistaxis or for any other reason. For patients requiring an inpatient admission, the length and reason for admission were recorded.

### Analysis

All statistical calculations were done using SAS (version 9.3). Categorical variables were summarized using frequency counts and percentages, while continuous variables were summarized using the mean (SD) or median (IQR), as appropriate. Where necessary, initial testing for associations between categorical variables was done using either chi-square or Fisher’s Exact tests. Modeling of categorical outcomes was done using logistic regression.

## Results

### Characteristics of study subjects

A total of 419 visits to the ED with a primary diagnosis of epistaxis occurred from January 2012 to May 2014. Sixty-six visits were excluded from this analysis, reasons for exclusion are shown in Fig. 1. Overall, 353 anterior epistaxis cases were included in this study; the demographics and comorbidities are summarized in Table 1. The individuals included in this study had a mean age of 70 and 49 % were women. A large proportion (61 %) of the patients were on some type of anticoagulant or antiplatelet medication. Of the comorbidities recorded, hypertension, diabetes, coronary artery disease, atrial fibrillation, did not have a statistically significant impact on treatment failure (*p* > 0.05).

**Table 1:** Patient demographics

Characteristic	Value
Age mean y (range),	70 (14–97)
Sex no. (%)	
Male	180 (51)
Female	173 (49)
Comorbidities N (%)	
Hypertension	198 (56)
Diabetes	67 (19)
CAD^a^	97 (28)
Afib^b^	94 (27)
HHT^c^	3 (1)
Other blood disorders	12 (3)
AC/AP^d^ medication use	217 (62)

^a^Coronary artery disease

^b^Atrial fibrillation

^c^Hereditary hemorrhagic telangiectasia

^d^Anticoagulation or antiplatelet

### Main results

The outcome of each treatment is summarized in Table 2. In all, the overall primary treatment failure rate was 26 % (91 patients) and in total 26.6 % (94 patients) returned to the ED for a scheduled follow-up after discharge from the ED. Of the individuals requiring follow-up, 89 (95 %) returned for packing removal (53 patients had Merocel® packing), in 3 (3.1 %) patients packing was left in situ at the follow up visit and 2 (2.1 %) patients attended the follow up visit even though their packing had fallen out on its own before their appointment. Of the 94 patients requiring follow up, 22 (23 %) required further intervention (10 patients with Merocel® packing) for epistaxis at the time packing removal. There was no difference in bleeding rates post pack removal between the different types of packing.

**Table 2:** Treatment outcomes for management of anterior epistaxis

Treatment	N (%)	Failure N (%)
Silver nitrate	122 (35)	24 (20)
Merocel	92 (26)	24 (26)
No treatment	54 (15)	11 (20)
Other packing^a^	45 (13)	19 (42)
Other^b^	23 (6)	3 (13)
Nasal clip	17 (5)	10 (59)

^a^Other packing included non-dissolvable anterior packs the majority being Vaseline gauze packing

^b^Other included surgicel, decongestant with topical anesthetic alone

When silver nitrate was compared to petroleum gauze packing, those in silver nitrate group were less likely to fail (OR 0.335, 95 % CI 0.160–0.703 *p* = 0.0038). When silver nitrate was compared to Merocel® packing, the odds of recurrence were lower with silver nitrate than with Merocel® (OR 0.694, 95 % CI 0.364–1.322, *p* = 0.27), however this was not statistically significant.

When evaluating potential risk factors for the development of epistaxis, anticoagulation was identified from the patient characteristics, through logistical regression. The type of anticoagulant or antiplatelet medication individuals in the study were receiving is summarized in Table 3. Given the large variety of anticoagulation and antiplatelet medications, they were grouped into 3 categories for analysis as seen in Table 4. Overall, 61 % of the individuals were on at least one antiplatelet or anticoagulant medication. Of those not on any anticoagulant or antiplatelet agent, the failure rate for anterior epistaxis treatment was 18 %. In contrast, for individuals on any anticoagulant/antiplatelet agent the failure rate was 30 %. There was a statistically significant association between the use of anticoagulant/antiplatelet medication and the recurrence of epistaxis (*p* = 0.0119). 73 % of all patients who failed treatment were on at least one antiplatelet or anticoagulant medication.

**Table 3:** Types of anticoagulation (AC)/antiplatelet (AP) medications used by patient population

Medication	N (%)
Any AC/AP	217 (62)
ASA	122 (34)
Coumadin	78 (23)
Rivaroxaban	14 (4)
Dabigatran	4 (1)
Apixaban	4 (1)
Clopidogrel	33 (9)
Ticagrelor	2 (1)
Other anticoagulant	7 (2)

**Table 4:** Outcomes of treatment success and failure based on anticoagulation/antiplatelet use profile

Anticoagulant/Antiplatelet	N	Failure N (%)
None	136	25 (18)
Any anticoagulant/antiplatelet	217	66 (30)
ASA only	85	28 (33)
Other regimen	132	38 (29)

## Discussion

Overall there were 353 cases of anterior epistaxis analyzed in this study for outcomes of treatment received in the ED. Silver nitrate cautery was the most popular modality used accounting for 35 % of initial treatment. However, the treatment of anterior epistaxis proved to be quite variable with Merocel®, petroleum gauze packing/other packing or a nasal clip commonly being used.

The group of patients who received no treatment at the ED was not used as a control to compare other treatment modalities given those patients not requiring treatment had stopped bleeding when seen by the ED physician or they did not have a bleeding episode of such a severity that it required any treatment. It would be an unfair comparison due to the inherent clinical difference in epistaxis severity. When the silver nitrate group was compared to the petroleum gauze packing, those in silver nitrate group were less likely to fail (*p* = 0.0038).

In this cohort, silver nitrate treatment had the lowest rate of treatment failure (20 %) of the most utilised treatment modalities and it also had the added benefit of not requiring an additional routine ED visit, as non-dissolvable packing did. Selection bias may have affected this observation as silver nitrate may have been used by ED physicians only in less severe cases. Other literature has described good success rates for anterior dissolvable packing [3, 4, 11, 12] and surgical techniques [3], however the number of individuals receiving these treatments in our cohort were too small for analysis.

Epistaxis management, as with any medical condition, should be tailored to the patient and the clinical situation [8]. In this study most patients with anterior epistaxis received successful management with silver nitrate cautery or Merocel® packing being the most commonly used modalities. Silver nitrate was particularly advantageous as it showed promising results insofar as treatment success without a need for follow-up. However, in these cases the site of bleeding was identifiable on anterior rhinoscopy examination and amenable to cautery with silver nitrate. This is in keeping with other studies which have shown that when the source of bleeding in epistaxis is identifiable chemical cautery has excellent success in the treatment of anterior epistaxis [2, 8–10].

Exploring the reasons for treatment failure, the use of blood thinners is largely believed to have an effect. In our study it was found that being on any anticoagulant or antiplatelet agent, including ASA, significantly increased the odds of recurrence after discharge from the ED (*p* = 0.0106). The rate of treatment failure in patients on any anticoagulant/antiplatelet agent was 30 %, in ASA alone was 33 % and in another regimen was 29 %, these were significantly greater than the failure rate of 18 % seen in the individuals not on any such therapy (*p* < 0.0119).

As with any study, this study has some limitations. The population size studied was not large enough to accurately comment on less commonly used forms of management for anterior epistaxis. Similarly, there was no data or rating on the severity of epistaxis on arrival to the ED that, in the end, may have affected physician treatment selection and also affected recurrence. This may confound the relationship between the treatment modality used and outcomes. At the institution of this study, patients presenting acutely with anterior epistaxis are seen first by an emergency physician, who may or may not utilise nasal endoscopy if the bleeding site is not readily identified on anterior rhinoscopy. Similarly, a standardized approach to patient evaluation prior to treatment selection was not utilised for the patients in this series. A standard approach to patient evaluation for anterior epistaxis such as the application of a topical decongestant/vasoconstrictor and analgesia prior to assessment for a bleeding site is needed. Given that the decision to use cautery requires visualization of the bleeding site the choice between packing and cautery for an ED physician may then have been affected. Further, there may also have been patients who were lost to follow up due to ED visits at other locations in the case of re-bleeding. Despite the limitations in this study, the large patient population allowed for informative evaluation of the treatment data.

## Conclusions

In summary, the current practices for the treatment of anterior epistaxis in the ED are quite variable. There are many modalities currently in use and there is not yet an accepted evidence-based recommendation to help guide treatment decisions. Looking at the four most common modalities used to treat anterior epistaxis in the ED from this study, the use of silver nitrate appears to be an effective management option taking into account the time and resources used for any other modality necessitating a patient to return to the ED. This suggests that if the anterior site of bleeding is identifiable, it is likely amenable to chemical cautery, silver nitrate be the first line treatment. However, due to limitations of the study, and that there was no grading system to identify epistaxis severity, a recommendation of silver nitrate cautery for all occurrences of anterior epistaxis cannot be given at this time. Further study is needed to determine the most efficacious treatment modality based on epistaxis severity.
